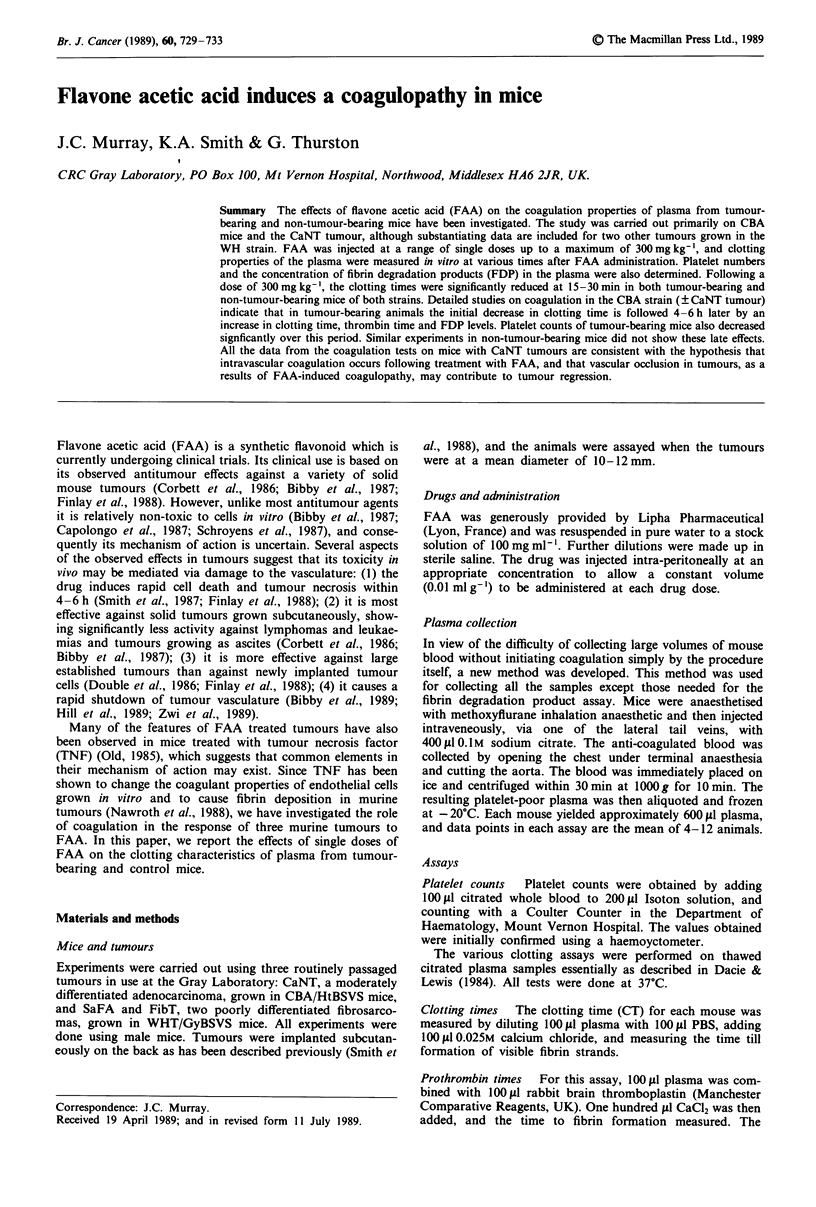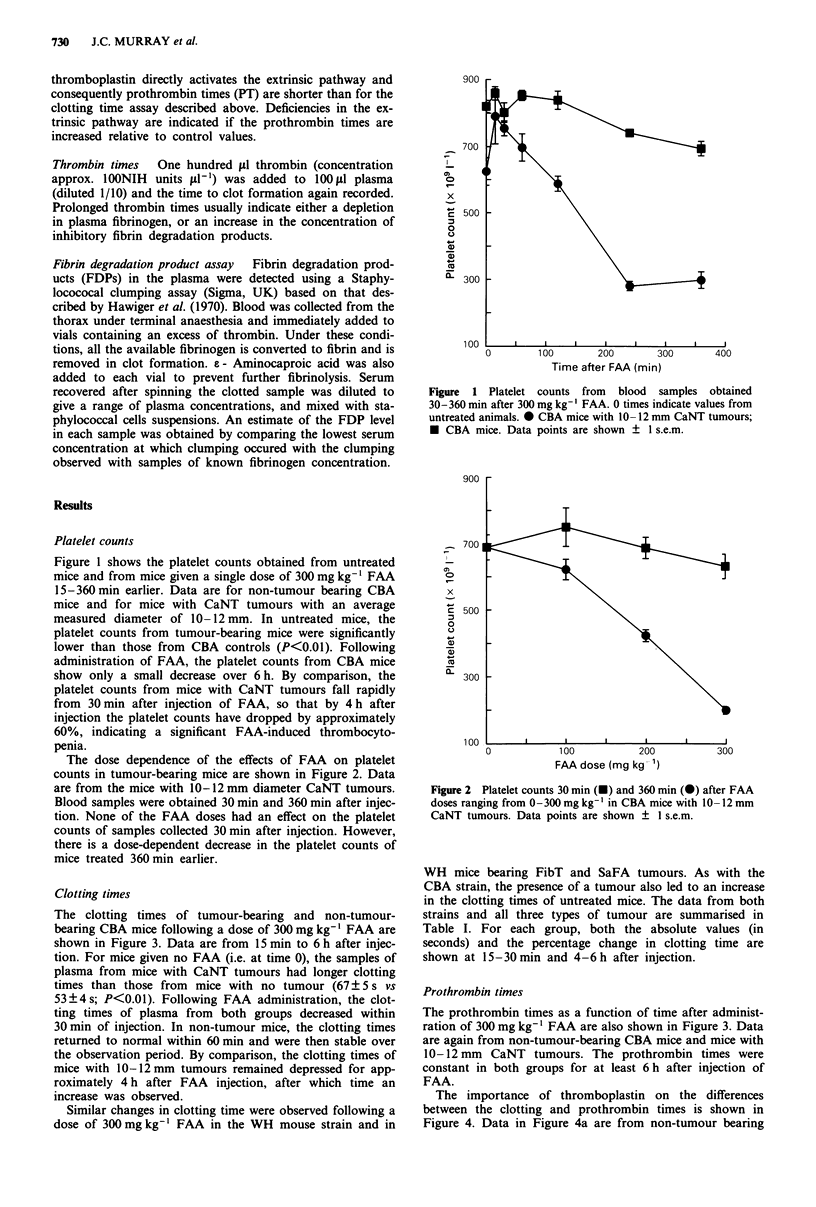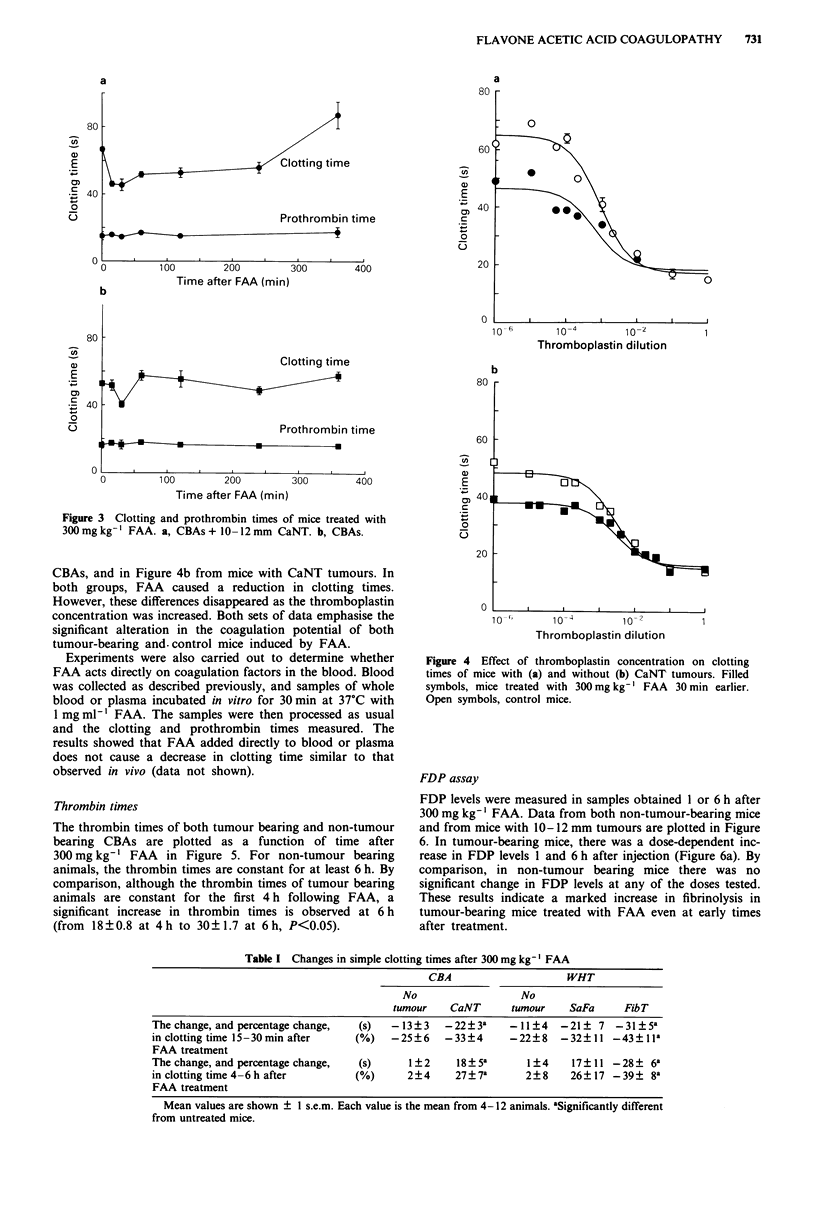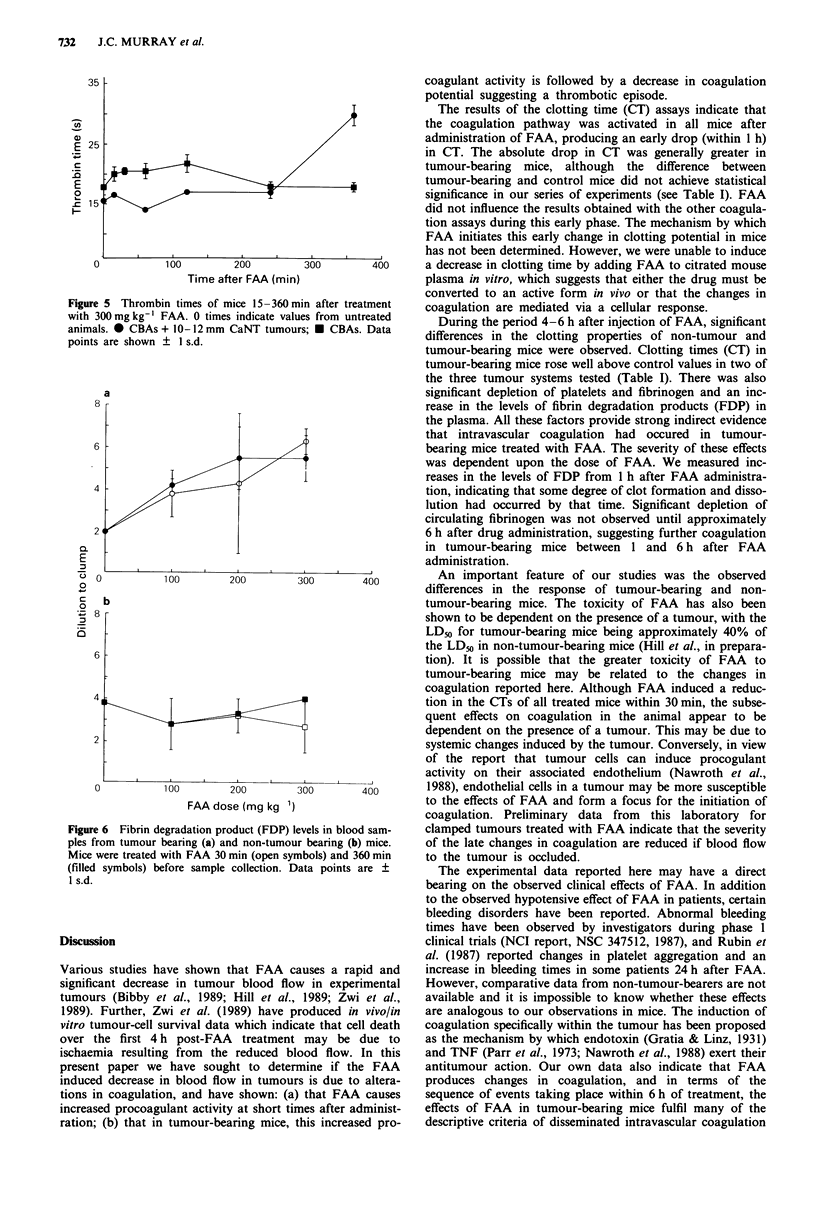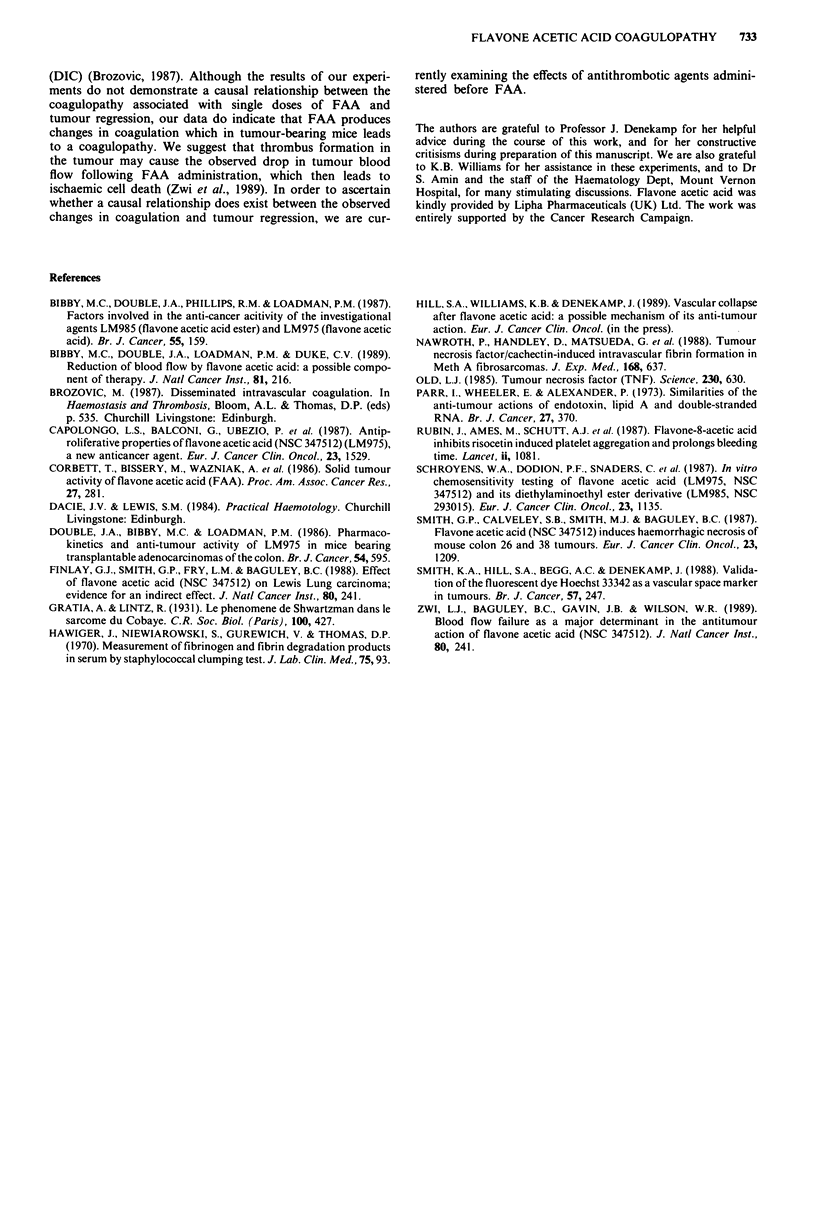# Flavone acetic acid induces a coagulopathy in mice.

**DOI:** 10.1038/bjc.1989.348

**Published:** 1989-11

**Authors:** J. C. Murray, K. A. Smith, G. Thurston

**Affiliations:** CRC Gray Laboratory, Mt Vernon Hospital, Northwood, Middlesex, UK.

## Abstract

The effects of flavone acetic acid (FAA) on the coagulation properties of plasma from tumour-bearing and non-tumour-bearing mice have been investigated. The study was carried out primarily on CBA mice and the CaNT tumour, although substantiating data are included for two other tumours grown in the WH strain. FAA was injected at a range of single doses up to a maximum of 300 mg kg-1, and clotting properties of the plasma were measured in vitro at various times after FAA administration. Platelet numbers and the concentration of fibrin degradation products (FDP) in the plasma were also determined. Following a dose of 300 mg kg-1, the clotting times were significantly reduced at 15-30 min in both tumour-bearing and non-tumour-bearing mice of both strains. Detailed studies on coagulation in the CBA strain (+/- CaNT tumour) indicate that in tumour-bearing animals the initial decrease in clotting time is followed 4-6 h later by an increase in clotting time, thrombin time and FDP levels. Platelet counts of tumour-bearing mice also decreased significantly over this period. Similar experiments in non-tumour-bearing mice did not show these late effects. All the data from the coagulation tests on mice with CaNT tumours are consistent with the hypothesis that intravascular coagulation occurs following treatment with FAA, and that vascular occlusion in tumours, as a results of FAA-induced coagulopathy, may contribute to tumour regression.


*Br. J. Cancer* (1989), **60**, 729–733 © The Macmillan Press Ltd., 1989

### Flavone acetic acid induces a coagulopathy in mice

J.C. Murray, K.A. Smith & G. Thurston

*CRC Gray Laboratory, PO Box 100, Mt Vernon Hospital, Northwood, Middlesex HA6 2JR, UK.*

**Summary** The effects of flavone acetic acid (FAA) on the coagulation properties of plasma from tumour-bearing and non-tumour-bearing mice have been investigated. The study was carried out primarily on CBA mice and the CaNT tumour, although substantiating data are included for two other tumours grown in the WH strain. FAA was injected at a range of single doses up to a maximum of $300\,mg\,kg^{-1}$, and clotting properties of the plasma were measured *in vitro* at various times after FAA administration. Platelet numbers and the concentration of fibrin degradation products (FDP) in the plasma were also determined. Following a dose of $300\,mg\,kg^{-1}$, the clotting times were significantly reduced at 15–30 min in both tumour-bearing and non-tumour-bearing mice of both strains. Detailed studies on coagulation in the CBA strain (±CaNT tumour) indicate that in tumour-bearing animals the initial decrease in clotting time is followed 4–6 h later by an increase in clotting time, thrombin time and FDP levels. Platelet counts of tumour-bearing mice also decreased signficantly over this period. Similar experiments in non-tumour-bearing mice did not show these late effects. All the data from the coagulation tests on mice with CaNT tumours are consistent with the hypothesis that intravascular coagulation occurs following treatment with FAA, and that vascular occlusion in tumours, as a results of FAA-induced coagulopathy, may contribute to tumour regression.

Flavone acetic acid (FAA) is a synthetic flavonoid which is currently undergoing clinical trials. Its clinical use is based on its observed antitumour effects against a variety of solid mouse tumours (Corbett *et al.*, 1986; Bibby *et al.*, 1987; Finlay *et al.*, 1988). However, unlike most antitumour agents it is relatively non-toxic to cells *in vitro* (Bibby *et al.*, 1987; Capolongo *et al.*, 1987; Schroyens *et al.*, 1987), and consequently its mechanism of action is uncertain. Several aspects of the observed effects in tumours suggest that its toxicity *in vivo* may be mediated via damage to the vasculature: (1) the drug induces rapid cell death and tumour necrosis within 4–6 h (Smith *et al.*, 1987; Finlay *et al.*, 1988); (2) it is most effective against solid tumours grown subcutaneously, showing significantly less activity against lymphomas and leukaemias and tumours growing as ascites (Corbett *et al.*, 1986; Bibby *et al.*, 1987); (3) it is more effective against large established tumours than against newly implanted tumour cells (Double *et al.*, 1986; Finlay *et al.*, 1988); (4) it causes a rapid shutdown of tumour vasculature (Bibby *et al.*, 1989; Hill *et al.*, 1989; Zwi *et al.*, 1989).

Many of the features of FAA treated tumours have also been observed in mice treated with tumour necrosis factor (TNF) (Old, 1985), which suggests that common elements in their mechanism of action may exist. Since TNF has been shown to change the coagulant properties of endothelial cells grown *in vitro* and to cause fibrin deposition in murine tumours (Nawroth *et al.*, 1988), we have investigated the role of coagulation in the response of three murine tumours to FAA. In this paper, we report the effects of single doses of FAA on the clotting characteristics of plasma from tumour-bearing and control mice.

### Materials and methods

### *Mice and tumours*

Experiments were carried out using three routinely passaged tumours in use at the Gray Laboratory: CaNT, a moderately differentiated adenocarcinoma, grown in CBA/HtBSVS mice, and SaFA and FibT, two poorly differentiated fibrosarcomas, grown in WHT/GyBSVS mice. All experiments were done using male mice. Tumours were implanted subcutaneously on the back as has been described previously (Smith *et al.*, 1988), and the animals were assayed when the tumours were at a mean diameter of 10–12 mm.

### *Drugs and administration*

FAA was generously provided by Lipha Pharmaceutical (Lyon, France) and was resuspended in pure water to a stock solution of $100\,mg\,ml^{-1}$. Further dilutions were made up in sterile saline. The drug was injected intra-peritoneally at an appropriate concentration to allow a constant volume ($0.01\,ml\,g^{-1}$) to be administered at each drug dose.

### *Plasma collection*

In view of the difficulty of collecting large volumes of mouse blood without initiating coagulation simply by the procedure itself, a new method was developed. This method was used for collecting all the samples except those needed for the fibrin degradation product assay. Mice were anaesthetised with methoxyflurane inhalation anaesthetic and then injected intraveneously, via one of the lateral tail veins, with 400 µl 0.1 M sodium citrate. The anti-coagulated blood was collected by opening the chest under terminal anaesthesia and cutting the aorta. The blood was immediately placed on ice and centrifuged within 30 min at 1000 *g* for 10 min. The resulting platelet-poor plasma was then aliquoted and frozen at −20°C. Each mouse yielded approximately 600 µl plasma, and data points in each assay are the mean of 4–12 animals.

### *Assays*

*Platelet counts* Platelet counts were obtained by adding 100 µl citrated whole blood to 200 µl Isoton solution, and counting with a Coulter Counter in the Department of Haematology, Mount Vernon Hospital. The values obtained were initially confirmed using a haemoyctometer.

The various clotting assays were performed on thawed citrated plasma samples essentially as described in Dacie & Lewis (1984). All tests were done at 37°C.

*Clotting times* The clotting time (CT) for each mouse was measured by diluting 100 µl plasma with 100 µl PBS, adding 100 µl 0.025 M calcium chloride, and measuring the time till formation of visible fibrin strands.

*Prothrombin times* For this assay, 100 µl plasma was combined with 100 µl rabbit brain thromboplastin (Manchester Comparative Reagents, UK). One hundred µl $CaCl_2$ was then added, and the time to fibrin formation measured. The

Correspondence: J.C. Murray.
Received 19 April 1989; and in revised form 11 July 1989.

thromboplastin directly activates the extrinsic pathway and consequently prothrombin times (PT) are shorter than for the clotting time assay described above. Deficiencies in the extrinsic pathway are indicated if the prothrombin times are increased relative to control values.

*Thrombin times* One hundred µl thrombin (concentration approx. 100NIH units $\mu l^{-1}$) was added to 100 µl plasma (diluted 1/10) and the time to clot formation again recorded. Prolonged thrombin times usually indicate either a depletion in plasma fibrinogen, or an increase in the concentration of inhibitory fibrin degradation products.

*Fibrin degradation product assay* Fibrin degradation products (FDPs) in the plasma were detected using a Staphylococcal clumping assay (Sigma, UK) based on that described by Hawiger *et al.* (1970). Blood was collected from the thorax under terminal anaesthesia and immediately added to vials containing an excess of thrombin. Under these conditions, all the available fibrinogen is converted to fibrin and is removed in clot formation. ε - Aminocaproic acid was also added to each vial to prevent further fibrinolysis. Serum recovered after spinning the clotted sample was diluted to give a range of plasma concentrations, and mixed with staphylococcal cells suspensions. An estimate of the FDP level in each sample was obtained by comparing the lowest serum concentration at which clumping occured with the clumping observed with samples of known fibrinogen concentration.

### Results

*Platelet counts*

Figure 1 shows the platelet counts obtained from untreated mice and from mice given a single dose of 300 mg $kg^{-1}$ FAA 15–360 min earlier. Data are for non-tumour bearing CBA mice and for mice with CaNT tumours with an average measured diameter of 10–12 mm. In untreated mice, the platelet counts from tumour-bearing mice were significantly lower than those from CBA controls ($P<0.01$). Following administration of FAA, the platelet counts from CBA mice show only a small decrease over 6 h. By comparison, the platelet counts from mice with CaNT tumours fall rapidly from 30 min after injection of FAA, so that by 4 h after injection the platelet counts have dropped by approximately 60%, indicating a significant FAA-induced thrombocytopenia.

The dose dependence of the effects of FAA on platelet counts in tumour-bearing mice are shown in Figure 2. Data are from the mice with 10–12 mm diameter CaNT tumours. Blood samples were obtained 30 min and 360 min after injection. None of the FAA doses had an effect on the platelet counts of samples collected 30 min after injection. However, there is a dose-dependent decrease in the platelet counts of mice treated 360 min earlier.

*Clotting times*

The clotting times of tumour-bearing and non-tumour-bearing CBA mice following a dose of 300 mg $kg^{-1}$ FAA are shown in Figure 3. Data are from 15 min to 6 h after injection. For mice given no FAA (i.e. at time 0), the samples of plasma from mice with CaNT tumours had longer clotting times than those from mice with no tumour ($67 \pm 5$ s *vs* $53 \pm 4$ s; $P<0.01$). Following FAA administration, the clotting times of plasma from both groups decreased within 30 min of injection. In non-tumour mice, the clotting times returned to normal within 60 min and were then stable over the observation period. By comparison, the clotting times of mice with 10–12 mm tumours remained depressed for approximately 4 h after FAA injection, after which time an increase was observed.

Similar changes in clotting time were observed following a dose of 300 mg $kg^{-1}$ FAA in the WH mouse strain and in

**Figure 1** Platelet counts from blood samples obtained 30–360 min after 300 mg $kg^{-1}$ FAA. 0 times indicate values from untreated animals. ● CBA mice with 10–12 mm CaNT tumours; ■ CBA mice. Data points are shown ± 1 s.e.m.

**Figure 2** Platelet counts 30 min (■) and 360 min (●) after FAA doses ranging from 0–300 mg $kg^{-1}$ in CBA mice with 10–12 mm CaNT tumours. Data points are shown ± 1 s.e.m.

WH mice bearing FibT and SaFA tumours. As with the CBA strain, the presence of a tumour also led to an increase in the clotting times of untreated mice. The data from both strains and all three types of tumour are summarised in Table I. For each group, both the absolute values (in seconds) and the percentage change in clotting time are shown at 15–30 min and 4–6 h after injection.

*Prothrombin times*

The prothrombin times as a function of time after administration of 300 mg $kg^{-1}$ FAA are also shown in Figure 3. Data are again from non-tumour-bearing CBA mice and mice with 10–12 mm CaNT tumours. The prothrombin times were constant in both groups for at least 6 h after injection of FAA.

The importance of thromboplastin on the differences between the clotting and prothrombin times is shown in Figure 4. Data in Figure 4a are from non-tumour bearing

**Figure 3** Clotting and prothrombin times of mice treated with 300 mg kg$^{-1}$ FAA. **a**, CBAs + 10–12 mm CaNT. **b**, CBAs.

CBAs, and in Figure 4b from mice with CaNT tumours. In both groups, FAA caused a reduction in clotting times. However, these differences disappeared as the thromboplastin concentration was increased. Both sets of data emphasise the significant alteration in the coagulation potential of both tumour-bearing and control mice induced by FAA.

Experiments were also carried out to determine whether FAA acts directly on coagulation factors in the blood. Blood was collected as described previously, and samples of whole blood or plasma incubated *in vitro* for 30 min at 37°C with 1 mg ml$^{-1}$ FAA. The samples were then processed as usual and the clotting and prothrombin times measured. The results showed that FAA added directly to blood or plasma does not cause a decrease in clotting time similar to that observed *in vivo* (data not shown).

### *Thrombin times*

The thrombin times of both tumour bearing and non-tumour bearing CBAs are plotted as a function of time after 300 mg kg$^{-1}$ FAA in Figure 5. For non-tumour bearing animals, the thrombin times are constant for at least 6 h. By comparison, although the thrombin times of tumour bearing animals are constant for the first 4 h following FAA, a significant increase in thrombin times is observed at 6 h (from 18±0.8 at 4 h to 30±1.7 at 6 h, $P<0.05$).

**Figure 4** Effect of thromboplastin concentration on clotting times of mice with (**a**) and without (**b**) CaNT tumours. Filled symbols, mice treated with 300 mg kg$^{-1}$ FAA 30 min earlier. Open symbols, control mice.

### *FDP assay*

FDP levels were measured in samples obtained 1 or 6 h after 300 mg kg$^{-1}$ FAA. Data from both non-tumour-bearing mice and from mice with 10–12 mm tumours are plotted in Figure 6. In tumour-bearing mice, there was a dose-dependent increase in FDP levels 1 and 6 h after injection (Figure 6a). By comparison, in non-tumour bearing mice there was no significant change in FDP levels at any of the doses tested. These results indicate a marked increase in fibrinolysis in tumour-bearing mice treated with FAA even at early times after treatment.

**Table I** Changes in simple clotting times after 300 mg kg$^{-1}$ FAA

| | | *CBA* | | *WHT* | | |
|---|---|---|---|---|---|---|
| | | *No tumour* | *CaNT* | *No tumour* | *SaFa* | *FibT* |
| The change, and percentage change, | (s) | −13±3 | −22±3[a] | −11±4 | −21± 7 | −31±5[a] |
| in clotting time 15–30 min after | (%) | −25±6 | −33±4 | −22±8 | −32±11 | −43±11[a] |
| FAA treatment | | | | | | |
| The change, and percentage change, | (s) | 1±2 | 18±5[a] | 1±4 | 17±11 | −28± 6[a] |
| in clotting time 4–6 h after | (%) | 2±4 | 27±7[a] | 2±8 | 26±17 | −39± 8[a] |
| FAA treatment | | | | | | |

Mean values are shown ± 1 s.e.m. Each value is the mean from 4–12 animals. [a]Significantly different from untreated mice.

**Figure 5** Thrombin times of mice 15–360 min after treatment with 300 mg kg$^{-1}$ FAA. 0 times indicate values from untreated animals. ● CBAs + 10–12 mm CaNT tumours; ■ CBAs. Data points are shown ± 1 s.d.

**Figure 6** Fibrin degradation product (FDP) levels in blood samples from tumour bearing (**a**) and non-tumour bearing (**b**) mice. Mice were treated with FAA 30 min (open symbols) and 360 min (filled symbols) before sample collection. Data points are ± 1 s.d.

### Discussion

Various studies have shown that FAA causes a rapid and significant decrease in tumour blood flow in experimental tumours (Bibby *et al.*, 1989; Hill *et al.*, 1989; Zwi *et al.*, 1989). Further, Zwi *et al.* (1989) have produced *in vivo/in vitro* tumour-cell survival data which indicate that cell death over the first 4 h post-FAA treatment may be due to ischaemia resulting from the reduced blood flow. In this present paper we have sought to determine if the FAA induced decrease in blood flow in tumours is due to alterations in coagulation, and have shown: (a) that FAA causes increased procoagulant activity at short times after administration; (b) that in tumour-bearing mice, this increased procoagulant activity is followed by a decrease in coagulation potential suggesting a thrombotic episode.

The results of the clotting time (CT) assays indicate that the coagulation pathway was activated in all mice after administration of FAA, producing an early drop (within 1 h) in CT. The absolute drop in CT was generally greater in tumour-bearing mice, although the difference between tumour-bearing and control mice did not achieve statistical significance in our series of experiments (see Table I). FAA did not influence the results obtained with the other coagulation assays during this early phase. The mechanism by which FAA initiates this early change in clotting potential in mice has not been determined. However, we were unable to induce a decrease in clotting time by adding FAA to citrated mouse plasma *in vitro*, which suggests that either the drug must be converted to an active form *in vivo* or that the changes in coagulation are mediated via a cellular response.

During the period 4–6 h after injection of FAA, significant differences in the clotting properties of non-tumour and tumour-bearing mice were observed. Clotting times (CT) in tumour-bearing mice rose well above control values in two of the three tumour systems tested (Table I). There was also significant depletion of platelets and fibrinogen and an increase in the levels of fibrin degradation products (FDP) in the plasma. All these factors provide strong indirect evidence that intravascular coagulation had occured in tumour-bearing mice treated with FAA. The severity of these effects was dependent upon the dose of FAA. We measured increases in the levels of FDP from 1 h after FAA administration, indicating that some degree of clot formation and dissolution had occurred by that time. Significant depletion of circulating fibrinogen was not observed until approximately 6 h after drug administration, suggesting further coagulation in tumour-bearing mice between 1 and 6 h after FAA administration.

An important feature of our studies was the observed differences in the response of tumour-bearing and non-tumour-bearing mice. The toxicity of FAA has also been shown to be dependent on the presence of a tumour, with the $LD_{50}$ for tumour-bearing mice being approximately 40% of the $LD_{50}$ in non-tumour-bearing mice (Hill *et al.*, in preparation). It is possible that the greater toxicity of FAA to tumour-bearing mice may be related to the changes in coagulation reported here. Although FAA induced a reduction in the CTs of all treated mice within 30 min, the subsequent effects on coagulation in the animal appear to be dependent on the presence of a tumour. This may be due to systemic changes induced by the tumour. Conversely, in view of the report that tumour cells can induce procoagulant activity on their associated endothelium (Nawroth *et al.*, 1988), endothelial cells in a tumour may be more susceptible to the effects of FAA and form a focus for the initiation of coagulation. Preliminary data from this laboratory for clamped tumours treated with FAA indicate that the severity of the late changes in coagulation are reduced if blood flow to the tumour is occluded.

The experimental data reported here may have a direct bearing on the observed clinical effects of FAA. In addition to the observed hypotensive effect of FAA in patients, certain bleeding disorders have been reported. Abnormal bleeding times have been observed by investigators during phase 1 clinical trials (NCI report, NSC 347512, 1987), and Rubin *et al.* (1987) reported changes in platelet aggregation and an increase in bleeding times in some patients 24 h after FAA. However, comparative data from non-tumour-bearers are not available and it is impossible to know whether these effects are analogous to our observations in mice. The induction of coagulation specifically within the tumour has been proposed as the mechanism by which endotoxin (Gratia & Linz, 1931) and TNF (Parr *et al.*, 1973; Nawroth *et al.*, 1988) exert their antitumour action. Our own data also indicate that FAA produces changes in coagulation, and in terms of the sequence of events taking place within 6 h of treatment, the effects of FAA in tumour-bearing mice fulfil many of the descriptive criteria of disseminated intravascular coagulation

(DIC) (Brozovic, 1987). Although the results of our experiments do not demonstrate a causal relationship between the coagulopathy associated with single doses of FAA and tumour regression, our data do indicate that FAA produces changes in coagulation which in tumour-bearing mice leads to a coagulopathy. We suggest that thrombus formation in the tumour may cause the observed drop in tumour blood flow following FAA administration, which then leads to ischaemic cell death (Zwi *et al.*, 1989). In order to ascertain whether a causal relationship does exist between the observed changes in coagulation and tumour regression, we are currently examining the effects of antithrombotic agents administered before FAA.

The authors are grateful to Professor J. Denekamp for her helpful advice during the course of this work, and for her constructive critisisms during preparation of this manuscript. We are also grateful to K.B. Williams for her assistance in these experiments, and to Dr S. Amin and the staff of the Haematology Dept, Mount Vernon Hospital, for many stimulating discussions. Flavone acetic acid was kindly provided by Lipha Pharmaceuticals (UK) Ltd. The work was entirely supported by the Cancer Research Campaign.

### References

BIBBY, M.C., DOUBLE, J.A., PHILLIPS, R.M. & LOADMAN, P.M. (1987). Factors involved in the anti-cancer acitivity of the investigational agents LM985 (flavone acetic acid ester) and LM975 (flavone acetic acid). *Br. J. Cancer*, **55**, 159.

BIBBY, M.C., DOUBLE, J.A., LOADMAN, P.M. & DUKE, C.V. (1989). Reduction of blood flow by flavone acetic acid: a possible component of therapy. *J. Natl Cancer Inst.*, **81**, 216.

BROZOVIC, M. (1987). Disseminated intravascular coagulation. In *Haemostasis and Thrombosis*, Bloom, A.L. & Thomas, D.P. (eds) p. 535. Churchill Livingstone: Edinburgh.

CAPOLONGO, L.S., BALCONI, G., UBEZIO, P. *et al.* (1987). Antiproliferative properties of flavone acetic acid (NSC 347512) (LM975), a new anticancer agent. *Eur. J. Cancer Clin. Oncol.*, **23**, 1529.

CORBETT, T., BISSERY, M., WAZNIAK, A. *et al.* (1986). Solid tumour activity of flavone acetic acid (FAA). *Proc. Am. Assoc. Cancer Res.*, **27**, 281.

DACIE, J.V. & LEWIS, S.M. (1984). *Practical Haemotology*. Churchill Livingstone: Edinburgh.

DOUBLE, J.A., BIBBY, M.C. & LOADMAN, P.M. (1986). Pharmacokinetics and anti-tumour activity of LM975 in mice bearing transplantable adenocarcinomas of the colon. *Br. J. Cancer*, **54**, 595.

FINLAY, G.J., SMITH, G.P., FRY, L.M. & BAGULEY, B.C. (1988). Effect of flavone acetic acid (NSC 347512) on Lewis Lung carcinoma; evidence for an indirect effect. *J. Natl Cancer Inst.*, **80**, 241.

GRATIA, A. & LINTZ, R. (1931). Le phenomene de Shwartzman dans le sarcome du Cobaye. *C.R. Soc. Biol. (Paris)*, **100**, 427.

HAWIGER, J., NIEWIAROWSKI, S., GUREWICH, V. & THOMAS, D.P. (1970). Measurement of fibrinogen and fibrin degradation products in serum by staphylococcal clumping test. *J. Lab. Clin. Med.*, **75**, 93.

HILL, S.A., WILLIAMS, K.B. & DENEKAMP, J. (1989). Vascular collapse after flavone acetic acid: a possible mechanism of its anti-tumour action. *Eur. J. Cancer Clin. Oncol.* (in the press).

NAWROTH, P., HANDLEY, D., MATSUEDA, G. *et al.* (1988). Tumour necrosis factor/cachectin-induced intravascular fibrin formation in Meth A fibrosarcomas. *J. Exp. Med.*, **168**, 637.

OLD, L.J. (1985). Tumour necrosis factor (TNF). *Science*, **230**, 630.

PARR, I., WHEELER, E. & ALEXANDER, P. (1973). Similarities of the anti-tumour actions of endotoxin, lipid A and double-stranded RNA. *Br. J. Cancer*, **27**, 370.

RUBIN, J., AMES, M., SCHUTT, A.J. *et al.* (1987). Flavone-8-acetic acid inhibits risocetin induced platelet aggregation and prolongs bleeding time. *Lancet*, **ii**, 1081.

SCHROYENS, W.A., DODION, P.F., SNADERS, C. *et al.* (1987). *In vitro* chemosensitivity testing of flavone acetic acid (LM975, NSC 347512) and its diethylaminoethyl ester derivative (LM985, NSC 293015). *Eur. J. Cancer Clin. Oncol.*, **23**, 1135.

SMITH, G.P., CALVELEY, S.B., SMITH, M.J. & BAGULEY, B.C. (1987). Flavone acetic acid (NSC 347512) induces haemorrhagic necrosis of mouse colon 26 and 38 tumours. *Eur. J. Cancer Clin. Oncol.*, **23**, 1209.

SMITH, K.A., HILL, S.A., BEGG, A.C. & DENEKAMP, J. (1988). Validation of the fluorescent dye Hoechst 33342 as a vascular space marker in tumours. *Br. J. Cancer*, **57**, 247.

ZWI, L.J., BAGULEY, B.C., GAVIN, J.B. & WILSON, W.R. (1989). Blood flow failure as a major determinant in the antitumour action of flavone acetic acid (NSC 347512). *J. Natl Cancer Inst.*, **80**, 241.